# The First In Vivo Study Shows That Gyrophoric Acid Changes Behavior of Healthy Laboratory Rats

**DOI:** 10.3390/ijms25126782

**Published:** 2024-06-20

**Authors:** Patrik Simko, Andrea Leskanicova, Maria Suvakova-Nunhart, Jan Koval, Nela Zidekova, Martina Karasova, Petra Majerova, Ludmila Verboova, Alzbeta Blicharova, Martin Kertys, Ivan Barvik, Andrej Kovac, Terezia Kiskova

**Affiliations:** 1 Institute of Biology and Ecology, Faculty of Science, Pavol Jozef Šafárik University in Kosice, 040 01 Kosice, Slovakia; patrik.simko1@student.upjs.sk (P.S.); andrea.stafurikova@student.upjs.sk (A.L.); jan.koval@lifem.sk (J.K.); 2 Institute of Chemistry, Faculty of Science, Pavol Jozef Šafárik University in Kosice, 040 01 Kosice, Slovakia; mariasuvakova892@gmail.com; 3 Biomedical Center Martin (BioMed), Jessenius Faculty of Medicine in Martin, Comenius University, 841 99 Bratislava, Slovakia; zidekova27@uniba.sk (N.Z.); martin.kertys@uniba.sk (M.K.); 4 Small Animal Clinic, University of Veterinary Medicine and Pharmacy in Kosic, 041 81 Kosice, Slovakia; martina.karasova@uvlf.sk; 5 Institute of Neuroimmunology, Slovak Academy of Sciences, 831 01 Bratislava, Slovakia; petra.majerova@savba.sk (P.M.); andrej.kovac@savba.sk (A.K.); 6 Institute of Pathology, Faculty of Medicine, Pavol Jozef Šafárik University in Kosice, 040 01 Kosice, Slovakia; ludmila.verboova@upjs.sk (L.V.); alzbeta.blicharova@upjs.sk (A.B.); 7 Institute of Physics, Faculty of Mathematics and Physics, Charles University, 110 00 Prague, Czech Republic; ivan.barvik@mff.cuni.cz

**Keywords:** gyrophoric acid, hippocampus, behavior, in vivo, rats, human serum albumin

## Abstract

Gyrophoric acid (GA), a lichen secondary metabolite, has attracted more attention during the last years because of its potential biological effects. Until now, its effect in vivo has not yet been demonstrated. The aim of our study was to evaluate the basic physicochemical and pharmacokinetic properties of GA, which are directly associated with its biological activities. The stability of the GA in various pH was assessed by conducting repeated UV-VIS spectral measurements. Microsomal stability in rat liver microsomes was performed using Ultra-Performance LC/MS. Binding to human serum albumin (HSA) was assessed using synchronous fluorescence spectra, and molecular docking analysis was used to reveal the binding site of GA to HSA. In the in vivo experiment, 24 Sprague-Dawley rats (Velaz, Únetice, Czech Republic) were used. The animals were divided as follows. The first group (*n* = 6) included healthy males as control intact rats (♂INT), and the second group (*n* = 6) included healthy females as controls (♀INT). Groups three and four (♂GA/*n* = 6 and ♀GA/*n* = 6) consisted of animals with daily administered GA (10 mg/kg body weight) in an ethanol-water solution per os for a one-month period. We found that GA remained stable under various pH and temperature conditions. It bonded to human serum albumin with the binding constant 1.788 × 10^6^ dm^3^mol^−1^ to reach the target tissue via this mechanism. In vivo, GA did not influence body mass gain, food, or fluid intake during the experiment. No liver toxicity was observed. However, GA increased the rearing frequency in behavioral tests (*p* < 0.01) and center crossings in the elevated plus-maze (*p* < 0.01 and *p* < 0.001, respectively). In addition, the time spent in the open arm was prolonged (*p* < 0.01 and *p* < 0.001, respectively). Notably, GA was able to pass through the blood–brain barrier, indicating its ability to permeate into the brain and to stimulate neurogenesis in the hilus and subgranular zone of the hippocampus. These observations highlight the potential role of GA in influencing brain function and neurogenesis.

## 1. Introduction

Lichens, fascinating symbiotic organisms composed of a photosynthetic partner (green algae or cyanobacteria) and a fungal partner, have been utilized for centuries in various fields, including medicine and industry. They serve as a vital food source for both animals and humans [1]. Lichens produce a plethora of primary and secondary metabolites. While primary metabolites are essential for proper growth of lichens, secondary metabolites protect plants against environmental factors [2]. Lichens contain 800–1050 secondary metabolites belonging to various groups, including aliphatic acids, anthraquinones, phenolic compounds, quinones, pulvinic acid derivatives, steroids, terpenes, and xanthones [2,3]. Thus, they are treasure troves of natural compounds with pharmacological potential, encompassing antimicrobial, antiproliferative, cytotoxic, and antioxidant effects [4,5,6,7,8].

Among these compounds, gyrophoric acid (GA) stands out as a remarkable natural substance produced by several lichen species, prominently found in 31 out of 33 lichen species within the genus *Umbilicaria* [9]. It has a wide range of positive biological properties, including anticancer and antimicrobial effects, and is used in industrial sectors, such as the production of dyes [6,10,11,12]. Chemically, GA is a polyphenolic depside with three aromatic rings of orselinic acid linked by ester bonds [13]. The aromatic rings in these phenolic compounds allow them to scavenge free radicals, giving GA its antioxidant properties. Moreover, these aromatic rings form a versatile structure that, along with hydroxyl and methyl groups, can anchor to the active sites of various enzymes and receptors. GA inhibits topoisomerase 1 activity in mammalian cells, leading to DNA damage and activation of the p53/p21 DNA damage pathway. It also inhibits the function of various other enzymes such as eukaryotic protein tyrosine phosphatase (PTP1B), zinc-dependent metalloprotease neprilysin (MME), bacterial urease, and enzymes responsible for glycation control [9].

The first study describing its cytotoxic activity was published in 2004 [14]. Since then, numerous studies have investigated the biological activities of GA in vitro, confirming its antiproliferative and cytotoxic effects in MM98, A431, HaCaT [15] A2780, HeLa, MCF-7, SK-BR-3, HT-29, HCT-116 p53(+/+), HCT-116 p53(−/−), HL-60, and Jurkat cells [16] or in 3D spheroid cells [17], or antibacterial and antifungal effects [17,18], or antioxidant effects [16,18,19,20,21,22,23].

Despite the wealth of knowledge regarding the biological activities of GA, no in vivo study has been conducted thus far. Therefore, the objective of our research was to assess the fundamental physicochemical and pharmacokinetic properties of GA and to carry out the first in vivo study on healthy animals.

## 2. Results

### 2.1. Physicochemical Properties of GA

#### 2.1.1. pH Properties and Stability

**Figure 1:**
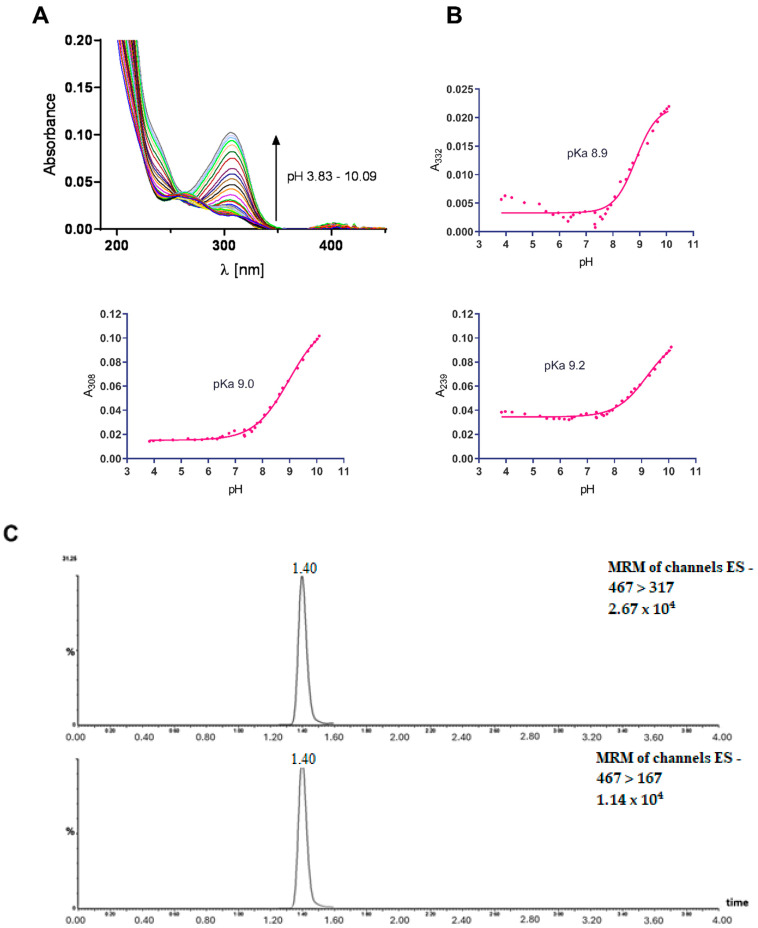
pH properties, stability, and absorbance. (**A**) Changes of gyrophoric acid (GA) 5 μM water solution UV-VIS spectra with pH ranging from 3.8 to 10.1. Spectra were measured in quartz cuvette with light path 1 cm in wavelength range from 184.5 to 550 nm. (**B**) Dependence of GA absorbance on pH at three wavelengths—239, 308 and 332 nm. Corresponding pKa values obtained by sigmoidal standard function were 9.2, 9.0, and 8.9, respectively. (**C**) Representative MRM ion-chromatograms of GA standard (31.25 ng/mL). MS/MS spectra for GA contained two ions at *m*/*z* 317 (**up**) and 167 (**down**), which were used for quantification and qualification transitions.

Measurements of GA water solution stability over time, as well as measurements of GA stability at different pH values (acidic, neutral, and alkaline), showed that GA is a stable molecule. Titration of a GA with acidified water solution from pH 3.8 to 10.1 showed UV-VIS spectral changes, providing data for pKa determination (Figure 1A). Significant spectral changes were observed at 239, 308, and 332 nm. The absorbance values corresponding to these wavelengths at different pH levels were fitted using a standard sigmoidal curve, and three pKa values of GA were determined for each wavelength (Figure 1B). The pKa values were approximately 9.

Experimentally determined pKa values of GA corresponded to values calculated by the chemicalize.com service (ChemAxon, Budapest, Hungary) The pKa value of a substance provides information about its acidic or basic behavior, and a pKa value of approximately 9 indicates that GA is more alkaline in nature. This suggests that a major microspecies of GA will be present in aqueous solutions in the pH range from 3.8 to approximately 9. Moreover, stability experiments conducted at room temperatures revealed that the prevalent GA remained stable under the given conditions.

#### 2.1.2. Microsomal Stability

Metabolic stability of GA was further assessed using rat liver microsomes (Figure 1C). The spectra showed that GA forms protonated molecular ions [M+H+] at *m*/*z* 467. MS/MS spectra for GA contained two ions at *m*/*z* 167 and 317, which were used for quantification and qualification transitions. The retention time of GA was 1.4 min. The calculated half-life was 33.7 min.

### 2.2. Binding to Human Serum Albumin

Human serum albumin (HSA) constitutes 60% of the total plasma protein, making it the most abundant plasma protein [24]. Understanding the interaction between GA and HSA is crucial for understanding the pharmacokinetics and pharmacodynamics of this bioactive substance [25]. The fluorescent properties of HSA are attributed to the amino acid residues Tyr, Trp, and Phe [26]. To investigate the binding of GA to HSA, we recorded the fluorescence emission spectra of HSA in the presence of various concentrations of GA. We observed a linear Stern–Volmer plot for GA. The parameters related to peak position or relative intensity are shown in Table 1, indicating the occurrence of only one type of quenching mechanism (static or dynamic) [27]. The value of kq 2.92811 × 10^13^ Lmol^−1^s^−1^ exceeded the maximum scatter collisional quenching constant (2 × 10^10^ M^−1^s^−1^) [28], suggesting that fluorescence quenching of HSA by GA is initiated by a static mechanism [27]. The binding constant of GA (1.788134 × 10^6^ dm^3^mol^−1^) fell within the optimal range, enabling significant transportation and distribution throughout the organism while also allowing the release of the compound once it reaches its target (Table 2). Furthermore, the value of *n* was approximately 1, indicating that only one independent class of binding sites is available for the studied derivatives on HSA [29]. Synchronous spectra revealed no shift in the maximum wavelength observed for Trp or Tyr residues, implying that their microenvironment polarity remained unaffected by the binding of GA to HSA. The curve for ∆λ = 60 nm was lower than that for ∆λ = 15 nm for GA, suggesting that Trp plays a crucial role in the fluorescence quenching of HSA [30]. The excitation of HSA in the presence of GA at 295 nm confirmed this observation [31]. The standard Gibbs free energy of the HSA–GA interaction had a negative value, indicating the spontaneity of the process. Conversely, the positive values of the standard enthalpy and entropy, along with changes in the quenching constant with temperature, indicate that hydrophobic interactions play a significant role in HSA–GA complex formation [32,33].

**Figure 2:**
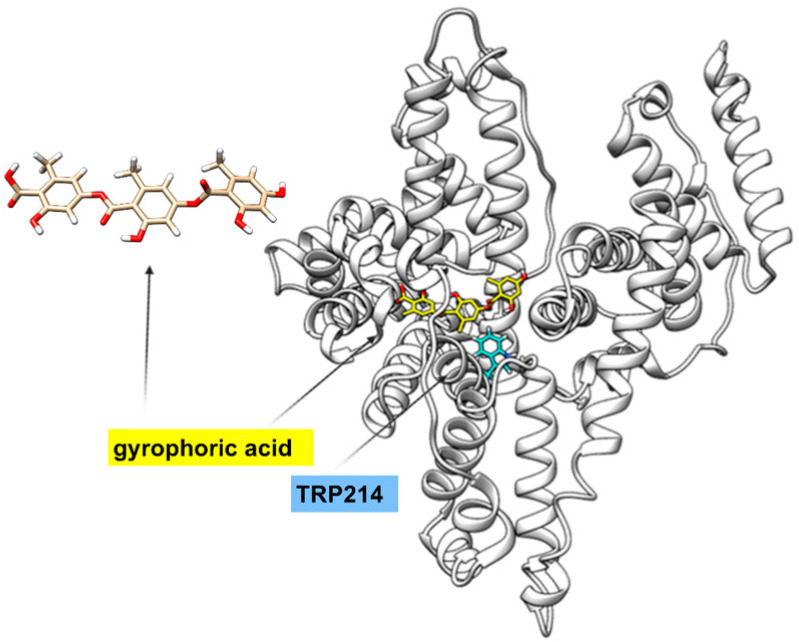
Binding to human serum albumin. Binding of GA (3D structure) near the binding site TRP214 of HSA (data from docking analysis).

The 3D fluorescence spectra provide information about the fluorescence of the protein as well as the changes in HSA conformation caused by drugs [34]. HSA has two fluorescence peaks. Peak 1 (λex/em = 280/342 nm/nm) primarily reflects the behavior of Trp or Tyr residues of HSA (fluorescence from Phe residues is negligible), whereas peak 2 (λex/em = 230/332 nm/nm) represents the behavior of polypeptide backbone structures [35]. The addition of GA reduced the fluorescence intensity of both the peaks. Peak 1 intensity decreased from 192 to 113 with a 2 nm hypsochromic shift, indicating GA interaction with Tyr and Trp residues of HSA. Peak 2 intensity decreased from 295 to 59 with a 7 nm Stokes shift, suggesting that GA caused changes in the polypeptide backbone conformation and secondary structure of HSA. GA binds to the TRP214 binding site in HSA (Figure 2).

### 2.3. In Vivo Experiments

#### 2.3.1. Body Mass Gain, Food, and Fluid Intake of Laboratory Animals

**Figure 3:**
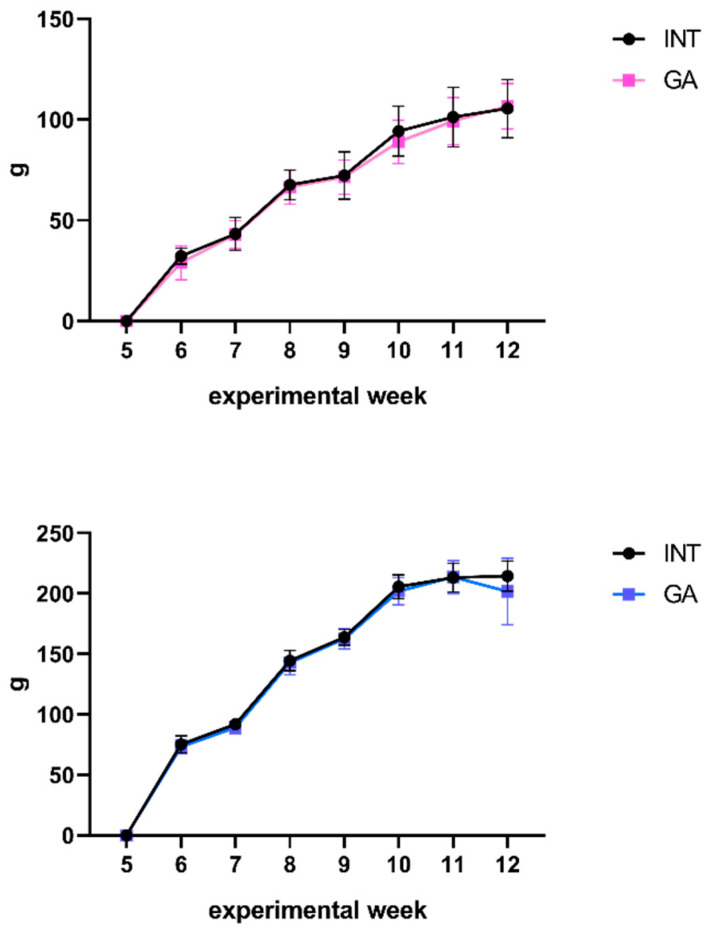
Body mass gain. Body mass gain in male (blue color) and female (pink color) animals in the INT and GA groups.

During the experiment, the rats were weighed weekly to monitor their body mass gain, a crucial parameter that may serve as an early indicator of the underlying pathology in the organism. After GA administration, no significant changes were observed, indicating that GA had no effect on weight gain (Figure 3). Similarly, no changes were observed in food or fluid intake (Table 3).

#### 2.3.2. Behavioral Analysis

**Figure 4:**
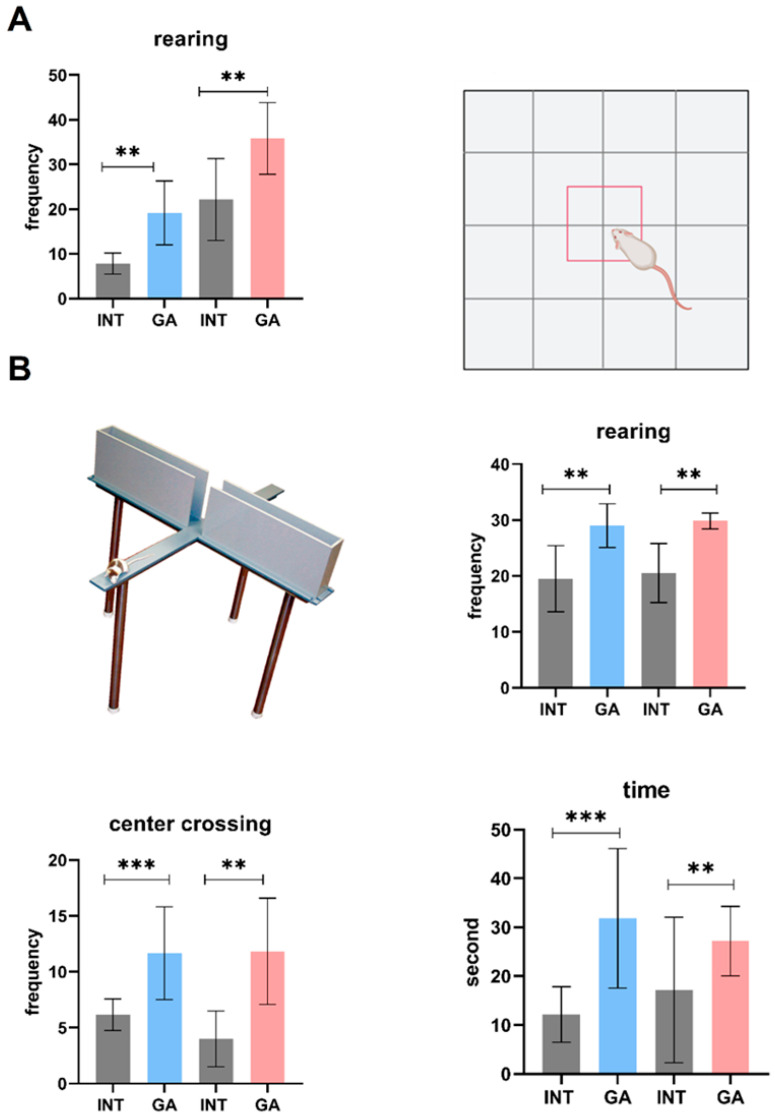
Behavioral analyses. (**A**) Open field test. (**B**) Elevated plus-maze. Data are expressed as mean ± SD. Significance vs. INT is given as ** *p* < 0.01, and *** *p* < 0.001, respectively. Rearing and center crossing are expressed as counts; time spent in open arms is expressed in seconds.

To monitor the basic forms of behavior, the open field test (OFT) and elevated plus-maze (EPM) test were selected. In the OFT, we observed increased rearing activity in both GA groups compared to the controls (*p* < 0.01) (Figure 4A). However, other parameters remained unchanged (Table S1).

We observed a significant increase in EPM for center crossings in the experimental group of GA males and females compared to the INT groups (*p* < 0.001 and *p* < 0.01, respectively). A significant prolongation was also recorded in the time spent in the open arms in both GA groups when compared to the INT group (*p* < 0.001 and *p* < 0.01, respectively). Similarly, rearing activity increased in the GA-treated groups (*p* < 0.05 and *p* < 0.01, respectively) (Figure 4B).

#### 2.3.3. Blood Analysis and Liver Histopathology

**Figure 5:**
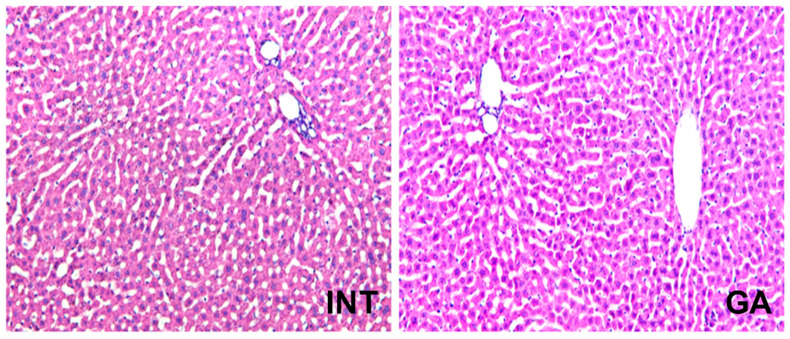
Blood analysis and liver. Representative photomicrographs of liver histopathology (200×): liver of INT control rats and GA-treated rats showing normal histology.

There was a significant decrease in both monocytes (MON) in males (*p* < 0.01) and females (*p* < 0.05), as well as granulocytes (GRA) in both sexes (*p* < 0.01) (Table 4). Furthermore, a significant decrease in ALT was noted in males (*p* < 0.05) and females (*p* < 0.01). Additionally, Table 5 shows a concurrent decrease in the levels of Crea and Urea in the groups treated with GA. None of those changes were seen during liver histopathology (Figure 5).

#### 2.3.4. Changes in Blood Metabolome

**Figure 6:**
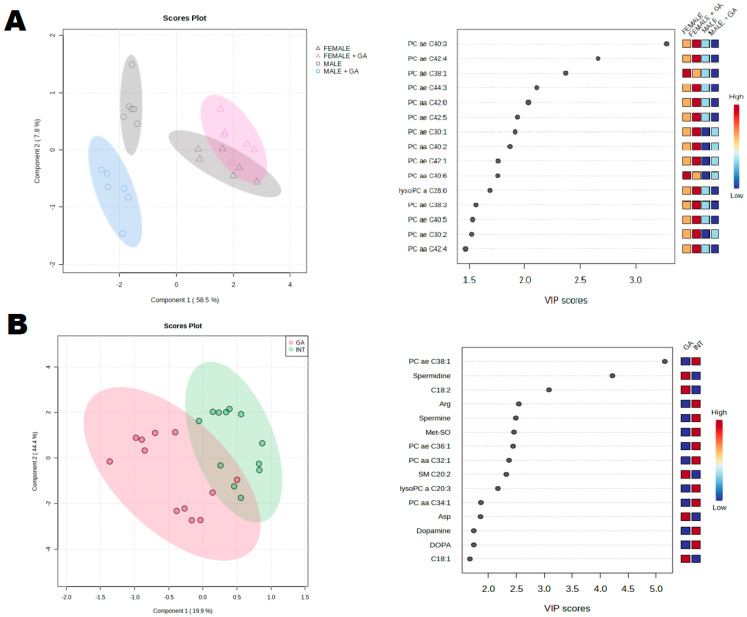
Blood metabolomics analysis. (**A**) Partial least squares-discrimination analysis (PLS-DA) of selected metabolites in male and female INT and GA-treated animals (**left side**). In the graphical output, 95% confidence ellipses for specific groups are included. Variable importance in projection (VIP) plot, calculated from the PLS-DA method, displays the top 15 most important metabolite features identified by PLS-DA (**right side**). Boxes on the right indicate the relative concentration of the corresponding metabolite in the blood in descending order of importance. VIP is a weighted sum of squares of the PLS-DA loadings considering the amount of explained Y-variable in each dimension. (**B**) Partial least squares-discrimination analysis (PLS-DA) of selected metabolites in INT and GA-treated animals, sex-independently (**left side**). In the graphical output, 95% confidence ellipses for specific groups are included. Variable importance in projection (VIP) plot, calculated from the PLS-DA method, displays the top 15 most important metabolite features identified by PLS-DA (**right side**). Boxes on the right indicate the relative concentration of the corresponding metabolite in the blood in descending or-der of importance. VIP is a weighted sum of squares of the PLS-DA loadings considering the amount of explained Y-variable in each dimension. The most important features have VIP values of >1.5.

Figure 6A shows that the main differences in blood metabolomics were due to sex differences resulting from the basic physiology. However, to determine the metabolic pathways through which GA operates, it was necessary to analyze both sexes together. Using a PLS-DA analysis and a VIP score, the most important features with scores greater than 2.0 were identified (Figure 6B). These features included PC ae C38:1, Spermidine, C18:2, Arginine (Arg), Methionine-sulfoxid (Met-SO), PC ae C:36:1, PC aa C32:1, SM C20:2, lysoPC a C20:3, and Aspartate (Asp).

#### 2.3.5. Blood–Brain Barrier Permeability

When testing the permeability of the GA in the primary model, the permeability coefficient reached 0.037944 ± 0.009511 × 10^−3^ cm/min.

#### 2.3.6. Neurogenesis and Neuronal Level

**Figure 7:**
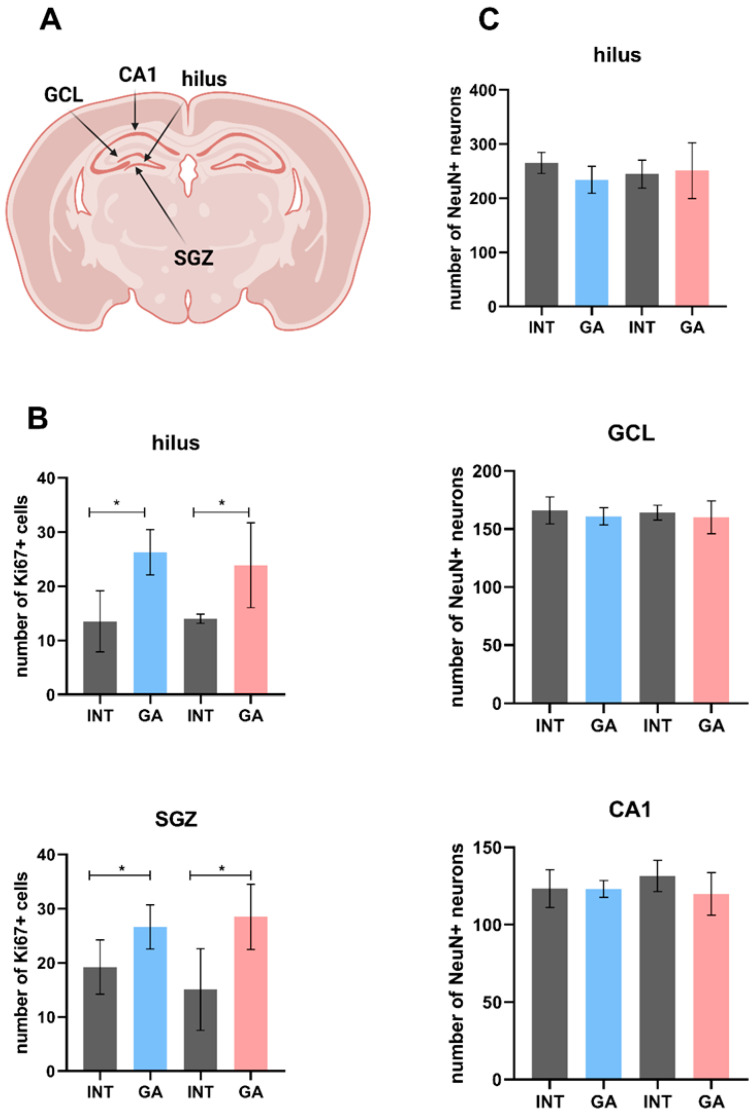
Hippocampus. (**A**) Schematic outline with selected hippocampal zones defined by the proliferation and postnatal neurogenesis, such as the hilus of dentate gyrus and subgranular zone (SGZ). The mature neurons were analyzed in the hilus part, granular cell layer (GCL), and CA1 zone. (**B**) The number of proliferative Ki67+ cells in the hilus and SGZ in individual groups of rats. (**C**) The number of mature NeuN+ neurons in the hilus and selected areas of GCL and CA1 zone in individual groups of rats. Values are given as arithmetic mean ± SD. Significance is indicated by * *p* ˂ 0.05.

To establish a connection between the observed behavioral changes in experimental animals, an investigation of the selected brain area was conducted (Figure 7A). Because hippocampal structure is known to be associated with the spatial orientation, short-term memory, and learning capacity, the level of proliferative active cells in this area was determined. As shown in Figure 7B, the number of Ki67+ cells increased significantly in both the hilus and subventricular zone (SVZ). On the other hand, the number of mature NeuN+ neurons remained unchanged (Figure 7C).

## 3. Discussion

Over the past five years, more than 60 scientific publications have been published exploring the potential biological effects of GA. However, none of these studies have been conducted under in vivo conditions. Our study, therefore, represents the first investigation into the effects of GA in vivo. The objective of our research was to analyze the fundamental behavioral patterns, hematological and biochemical parameters, and blood metabolome of healthy Sprague-Dawley rats. Our findings reveal that GA is a molecule with low solubility, particularly in alkaline environments, yet it remains stable across different pH conditions. It has the ability to bind to HSA, potentially facilitating its transportation to target tissues. The half-life of GA was observed to be approximately 33 min. Administering a dose of 10 mg/kg of GA did not affect the food and fluid intake, nor did it impact body mass gain of experimental animals. However, we did observe changes in certain behavioral patterns. Specifically, in the OFT, the rats exhibited increased rearing activity. Additionally, in the EPM, apart from increased rearing activity, the rats demonstrated heightened center crossings and time spent in the open arms of the maze. Concomitantly, GA promotes proliferative activity of hippocampal progenitors, indicating its neurogenic potential.

Drug delivery into the organism involves various administration routes, highlighting the importance of assessing the stability of xenobiotic under different pH conditions. As early as 1956, GA was recognized as a highly pH-sensitive molecule [36]. Nevertheless, our findings indicate that GA exhibits stability across a range of pH conditions.

HSA, as an abundant plasma protein, has garnered significant interest in the pharmaceutical industry due to its ability to bind a wide range of drugs, influencing their delivery and efficacy, and ultimately impacting the drugs’ pharmacokinetic and pharmacodynamics properties [37]. Our findings indicate that spontaneous hydrophobic interactions play a significant role in the formation of the HSA–GA complex. Moreover, our results demonstrate that GA exhibits optimal affinity for HSA, which is crucial for the distribution of xenobiotic through plasma proteins and their subsequent release in targeted tissues. It is likely that GA binds to a specific binding site on HSA, with docking analysis data suggesting that GA binds in proximity to the TRP214 binding site on HSA.

The synchronous spectra obtained from studying the interaction of GA with HSA suggest that there are no significant changes in the microenvironment polarity near the Trp and Tyr residues. However, the 3D fluorescence spectra present contrasting results. This disparity may be attributed to the nature of the 3D measurement, as the prolonged measurement time could potentially induce structural changes in GA within the aqueous environment, consequently affecting the measurement results.

Because of the lack of available GA amount, we were not able to find lethal and semi-lethal concentrations of this compound, which seems to be a limitation of our study. Due to the absence of prior in vivo studies involving GA, we opted to utilize a similar dosage that has been employed for other secondary metabolites, such as atranorin [38,39], or usnic acid [40]. Accordingly, GA was administered orally at a concentration of 10 mg/kg body weight on a daily basis for a duration of one month, as outlined in the Materials and Methods section. To assess potential health-related changes, we measured food and fluid intake, as well as body mass gain, which are crucial indicators. Due to a number of studies dealing with ethanol consumption, we missed out the control group treated with ethanol as a solvent, which is a limitation of our study.

Our findings indicate that GA had no discernible impact on food and fluid intake. Furthermore, there were no significant differences in body mass gain between the GA-treated group and the control group. Based on these results, we can infer that GA does not adversely affect body weight gain in both sexes.

Concomitantly with these results, we saw no substantial effects of GA on hematological parameters. The only noticeable parameters were depressed MON and GRA levels without any change of total count of WBC. It is important to note that a decrease in MON and GRA levels within the reference range is not considered clinically relevant. In healthy animals, these values can be as low as zero. Therefore, our results suggest that GA does not exert any negative impact on blood parameters, reinforcing its potential as a safe candidate in the field of herbal medicine.

From biochemical parameters, ALT was decreased. ALT is generally recognized as a specific indicator of hepatocellular damage. Elevated ALT activity typically signifies primary liver damage, which can be caused by factors such as drug-induced injury or secondary reactive hepatopathy associated with other organ or systemic diseases [41]. On the other hand, a decrease in ALT levels may be a promising indicator of liver parenchymal regeneration, especially if another drug with potential hepatotoxicity is used in therapy together with GA. A study of Park et al. (2019) revealed that low ALT was a significant independent predictor for liver-related mortality [42]. Additionally, two other parameters, urea and creatinine, showed decreased levels after GA treatment. Furthermore, the blood metabolome analysis revealed decreased levels of arginine, indicating the involvement of the urea cycle. Moreover, lipids from the phosphatidylcholine, lysophosphatidylcholine, and sphingomyelin groups were affected. These various mechanisms suggest ongoing processes within the liver. As GA is insoluble in majority of safe solvents, we have chosen ethanol as it has been shown to be safe at applied concentration [43,44]. Despite all of those facts, the liver remained histologically unchanged, suggesting the possible protective antioxidant effect of GA.

Considering the minimal alterations in hematological and biochemical parameters, we can conclude that administration of GA in the dose of 10 mg/kg to rats for a one-month period of time is safe and does not cause any changes in laboratory parameters that could be termed as an adverse effect of the therapy.

In behavioral tests, we monitored the animals to assess levels of various forms of behavior, such as locomotor and exploratory activity, comfort behavior, and anxiety. OFT is a test used to evaluate exploratory behavior and locomotor activity, as well as anxiety behavior in rodents [45]. In the OFT, we observed an increased frequency of rearing in both experimental groups compared to the INT groups. Exploratory rearing is one of the markers to evaluate the level of exploration of a novel environment [46]. Other parameters in this test, such as defecation, washing activity, time in the center, time in the periphery, travelled distance, and average speed, followed the INT groups analogously. Concomitantly, an increased rearing frequency after GA administration was observed in EPM. In addition to the aforementioned behaviors, we also observed an increased number of crosses through the center of the maze and an extended duration spent in the open arms of the maze. These observations suggest reduced anxiety-like behavior and increased exploratory tendencies in the tested animals. The travelled distance is an expression of locomotor activity in rodents [47], while time spent in open arms, frequency of defecation, and frequency of rearing are expressions of the level of depression in rodents [48]. Based on the EPM, we can evaluate the level of anxiety in rodents, which increased with decreasing time spent in the open arms of the maze by rodents and with lower number of passes through the center of the maze [49]. All those forms of behavior give indices that GA may depress anxiety behavior of the animals and sustains the natural exploration.

Because the changes in behavior are associated with the changes in brain structures, we focused also on the permeability coefficient of blood-brain-barrier transport. As shown, the permeability coefficient was 0.037944 ± 0.009511 × 10^−3^ cm/min. This value is very low as compared with for example propranolol [50] or sucrose [51]. As indicated before, the compounds with permeability coefficient *10^−6^ cm/s > 1.5 are classified as high permeation predicted, with Pe *10^−6^ cm/s < 1.5 classified as low permeation predicted [52]. For example, physodic acid from the extract of *Hypogymnia physodes* shows a permeability coefficient regarded as high (>1.5 × 10^−6^ cm/s) [53].

## 4. Materials and Methods

### 4.1. pH Properties and Stability

A 2 mM stock solution of GA (provided by RNDr. Michal Goga, PhD., UPJŠ Košice) was prepared by dissolving it in 96% ethanol and storing it in a dark environment at 8 °C. To create the working solution of GA, 5 μL of the stock solution was added to 2000 μL of distilled water. The stability of the GA solution in different pH conditions (acidic, neutral, and alkaline) was assessed by conducting repeated UV-VIS spectral measurements over a period of time. The pH measurements were conducted using a Hanna Instruments HI 2211 pH/ORP meter (Hanna Instruments, Woonsocket, RI, USA) equipped with a Hanna Instruments HI 1131 B electrode. UV-VIS spectra were acquired using a Specord S300 UV-VIS spectrometer (Analytik Jena AG, Jena, Germany) within the wavelength range of 184.5–550 nm. A quartz cuvette with a 1 cm light path was utilized for this purpose. The pH-related characteristics were investigated by observing pH-dependent alterations in the UV-VIS spectra of the GA aqueous solution, which was continuously agitated. The GA concentration in the working solution was maintained at 5 μM, and the spectra were recorded at a temperature of 22 °C. Initially, the aqueous GA solution underwent acidification through the addition of concentrated hydrochloric acid. The pH of the solution was adjusted to 3.8 initially. Subsequently, small increments of a 4 M NaOH aqueous solution were systematically introduced into the working solution within a cuvette, and both the pH and UV-VIS spectra were recorded. The titration process was concluded when the pH reached 10.1. Data obtained at wavelengths 239 nm, 308 nm, and 332 nm were subjected to analysis using GraphPad Prism 7.00 software. The pH curves were fitted using the sigmoidal standard curve model (Sigmoidal, 4PL, Warsaw, Poland) with X representing log(Concentration). To validate the results, the experimentally acquired data were compared with GA pKa values calculated using the Chemicalize.com web service, provided by © 2021 ChemAxon.

### 4.2. Microsomal Stability

The microsomal stability assay for insoluble compounds was performed as described by Di et al. [54]. Briefly, GA was solubilized in DMSO (CAS no.67-68-5, ≥99.8% purity, Sigma Aldrich, St. Louis, MO, USA). Concentrated rat liver microsomes (Thermo Fisher Scientific, Waltham, MA, USA) were mixed with pre-warmed phosphate buffer (100 mM), and GA was added. The reaction was terminated by addition of 20 mM NADPH (Merck Life Science, Bratislava, Slovakia). The mixture was incubated at 37 °C for up to 120 min, terminated by the addition of acetonitrile, and centrifuged (30,000× *g*/10 min). The supernatant was transferred into vials and analyzed using LC-MS/MS.

### 4.3. Ultra-Performance Liquid Chromatography Coupled to Tandem Mass Spectrometry

A Waters (Waters, Prague, Czechia) triple-quadrupole (Quattro premier XE) mass spectrometer with an electrospray ionization source in positive mode (ESI+) was used. The mass spectrometer was operated with the following parameters: capillary voltage, 3 kV; source temperature, 150 °C; desolvation temperature, 350 °C; cone gas, 50 L/h; and desolvation gas, 800 L/h. The source cone voltages and collision energies were manually optimized for each SRM transition. The chromatographic apparatus consisted of a Waters ACQUITY UPLC system with a binary gradient pump, autosampler, and column thermostat (Waters, Czechia). Chromatographic separation was performed on an ACQUITY UPLC BEH C18 (Thermo Fisher Scientific, USA) (2.1 × 50 mm, 1.7 μm particle size) column. The column temperature was maintained at 30 °C. Mobile phase A consisted of 0.1% formic acid in water. Mobile phase B consisted of 100% acetonitrile. The elution started at 20% B (0–0.5 min), increased to 90% B (0.5–2 min) to 90% B (2–3 min), returned to 20% B, and re-equilibrated at 3–4 min. The flow rate was 0.5 mL/min, and the injection volume was 5 μL. MassLynx software version 4.1 was used for instrument control, data acquisition, and analysis.

### 4.4. Binding to Human Serum Albumin

GA was dissolved in DMSO (CAS no.67-68-5, ≥99.8% purity, Sigma Aldrich, USA) to obtain a stock solution with a concentration of 0.7 µM. The stock solution was stored at −20 °C in the dark. Albumin from human serum (A1887, CAS no. 70024-90-7) and reagents for buffer solutions were obtained from Sigma Aldrich (USA).

The HSA stock solution was prepared by dissolving 20 × 10^−3^ g/mL in 150 × 10^−3^ M Tris-HCl-NaCl (pH 7.4, 22 °C). The concentration of the stock solution was determined by UV-Vis spectroscopy (Specord S300 UV VIS, Analytik Jena) using a quartz cuvette with an optical length of 1 cm. The extinction coefficient of HSA used in the calculation was ε280 = 35,700 M^−1^cm^−1^ [55]. The HSA stock solution was stored at 4–8 °C in the dark.

Fluorescence quenching spectra were acquired using a Varian Cary Eclipse spectrofluorimeter (Varian, Inc., Palo Alto, CA, USA) within the wavelength range of 290–500 nm. A quartz cuvette with a 1 cm optical path length was employed for the measurements. The excitation slit width was set to 5 nm, and the emission slit width was adjusted to 10 nm. An excitation wavelength of 280 nm was used, with an averaging time of 0.5 s and a data interval of 1 nm. The spectra were recorded at constant human serum albumin (HSA) concentrations (c = 4.0 × 10^−6^ M) while varying the concentrations of Glycyrrhetinic acid (GA) in the range of 0.4–3.5 × 10^−6^ M. These measurements were conducted in a 10 × 10^−3^ M Tris-HCl buffer solution at pH 7.4 and a temperature of 22 °C. The experiments were repeated at three different temperatures: 15 °C, 25 °C, and 37 °C. Fluorescence quenching spectra were obtained using a Varian Cary Eclipse spectrofluorimeter (Varian, Inc., USA) in the wavelength range of 290–500 nm using a quartz cuvette with an optical length of 1 cm. The excitation slit width was 5 nm, and the emission slit width was set at 10 nm. An excitation wavelength of 280 nm, averaging time of 0.5 s, and 1 nm data interval were used. Spectra were measured at fixed HSA concentrations (c = 4.0 × 10^−6^ M) and increasing concentrations of GA (0.4–3.5 × 10^−6^ M) in 10 × 10^−3^ M Tris-HCl buffer solution (pH 7.4, 22 °C). The measurements were performed at 15, 25, and 37 °C.

Synchronous fluorescence spectra were captured at two distinct scanning intervals, ∆λ, defined as ∆λ = λem − λex. The excitation wavelength ranged from 200 to 350 nm, and fluorescence emission was recorded at ∆ = 15 nm for tyrosine (Tyr) residues and ∆ = 60 nm for tryptophan (Trp) residues. The concentrations of human serum albumin (HSA) and the ligands matched those employed in the fluorescence spectral measurements. All measurements were conducted at a temperature of 25 °C.

The GA molecule was subjected to molecular docking within the binding site of HSA, originally occupied by Thyroxine. This binding site corresponds to the configuration observed in the original crystal structure with PDB identification number 1HK1 [56].

### 4.5. Experimental Design of In Vivo Experiments

**Figure 8:**
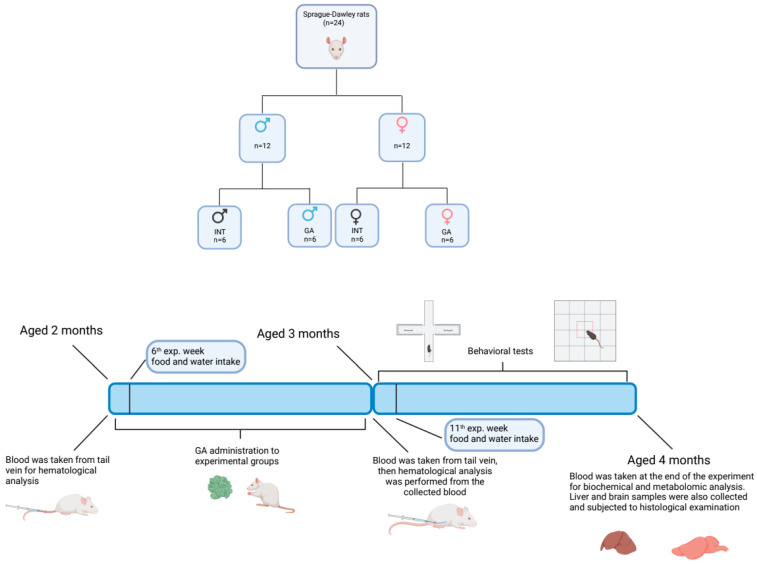
Experimental design.

Twenty-four Sprague-Dawley rats (Velaz, Czechia) were used in the experiment. The animals were maintained under standard vivarium conditions with a light regimen of 12:12 h, stable temperature (21–24 °C), and humidity (50–65%). The rats had free access to water and were fed with standard pellets (Velaz, Czechia) ad libitum. The animals were handled according to the guidelines established by Law Nos. 377 and 436/2012 of the Slovak Republic for the Care and Use of Laboratory Animals (approval numbers: Ro-2866/16-221, Ro-2219/19-221/3) and in accordance with ARRIVE guidelines. The animals were divided into four experimental groups. Each group consisted of six animals. The animals were divided according to sex into two control intact healthy groups (INT males and INT females) and two groups with daily administration of GA (GA males and GA females). GA at a dose of 10 mg/kg body weight (dissolved in 10% ethanol solution) was administered orally for a period of one month. Control rats were left undisturbed throughout the entire protocol, except for blood collection, behavioral testing, and regular cage cleaning, which was done for all animals by the same experimenter. Blood was collected from the tail vein before and after treatment for hematological analyses. Whole blood in K3EDTA tubes was analyzed using an automated veterinary hematology analyzer (MindrayBC 2800VET, Guangzhou, China). At the end of the experiment, blood was collected again (for biochemical and metabolomics analyses). Serum samples were stored at 4 °C, and biochemical analysis was performed over two consecutive days using an automated clinical chemistry analyzer (Cobas c 111, Roche, Basel, Switzerland) according to the manufacturer’s instructions (Figure 8).

### 4.6. Behavioral Analyses

The conduct of an organism mirrors its mental and physical condition. To assess animal behavior, two distinct behavioral assessments were employed to observe fundamental exploratory conduct: movement (open field test (OFT)) and levels of anxiety (elevated plus-maze (EPM)).

During the open field test (OFT), various parameters were measured, including locomotor activity and exploration. These measurements encompassed the total distance covered and the average speed of the animals, as well as the frequency of rearing. Additionally, anxiety levels were assessed by recording the number of center crossings, the time spent in the center versus the periphery, the number of defecation boluses, and the frequency of washing. At the commencement of each trial, the animals were positioned in the central area of the testing field, and each trial had a duration of 6 min. Throughout the test, the animals were continuously recorded using video footage, and the subsequent analysis of the results was conducted using Smart Junior software (ver. 3.0) (Panlab, Barcelona, Spain).

During the elevated plus-maze (EPM) test, various parameters were recorded, including locomotor activity (quantified by the number of center crossings), exploratory activity (measured by rearing frequency), and the level of anxiety (evaluated through time spent in open arms, the number of defecation events, and washing frequency). To initiate the test, the animals were positioned in the central square of the maze, and each trial had a duration of 6 min. For a detailed description, see [38].

### 4.7. Metabolomics

A total volume of 100 µL of blood was obtained from the tail vein at a single time point and collected into microtubes. The collected blood serum was then preserved by storing it at −80 °C. After thawing, the metabolites were analyzed using liquid chromatography-tandem mass spectrometry (LC-MS/MS) with the assistance of a Biocrates AbsoluteIDQ p180 kit (Biocrates Life Sciences AG, Innsbruck, Austria). Chromatographic separation was accomplished using an ultra-performance liquid chromatography system (ACQUITY UPLC I-Class, Waters, Czechia). This chromatography system was coupled with a triple quadrupole mass spectrometer (XEVO TQ-S, Waters, Czechia). Metabolites were extracted and quantified following the manufacturer’s guidelines, as previously [27,57,58]. The values were subjected to a log2 transformation to achieve normally distributed data and to stabilize the variance, consistent with the previous description [38].

### 4.8. The Blood–Brain Barrier Transport

Rat-mixed glial cell culture was prepared from the cerebral cortices of 0–2-day-old Sprague-Dawley rats as described previously [59]. Briefly, cerebral cortices were dissected, stripped of the meninges, and mechanically dissociated by repeated pipetting, followed by passage through a 20 µm nylon mesh. Cells were plated in 6-well plates pre-coated with poly-l-lysine (10 µg/mL; Sigma-Aldrich, USA) and cultivated in DMEM containing 10% FCS and 2 mM ultraglutamine (LONZA, Basel, Switzerland) at 37 °C and 5% CO_2_ in a humidified atmosphere. For further experiments, three-week-old glial cultures were used.

Primary rat brain endothelial cells were isolated as described previously [60]. Briefly, Sprague-Dawley rats were euthanized, and their whole brains were removed. Under sterile conditions, the brainstem and cerebellum were dissected, and the midbrain, white matter, and choroid plexus were removed. The remaining cortical tissue was removed from the meninges using dry Whatman paper. Tissue was homogenized on ice in DMEM-F12 (PAA Laboratories, Cölbe, Germany). Brain homogenate was centrifuged at 800× *g*/10 min at 4 °C. The supernatant was aspirated, and the pellet was re-suspended in pre-warmed digestion mix containing collagenase/dispase (Roche Diagnostics, Indianapolis, IN, USA) and DNase I (Sigma-Aldrich, USA). The tissue was incubated with the prepared digestion mix at 37 °C for 30 min with gentle shaking. Digested tissue was centrifuged at 800× *g*/10 min at 4 °C, and the pellet was re-suspended in 20% bovine serum albumin (BSA, Sigma-Aldrich, USA). The tissue was centrifuged at 1500× *g*/15 min at 4 °C to obtain pellets containing microvessels with a fraction of myelin and BSA at the top, which was centrifuged again. The microvessels were pooled, re-suspended in a pre-warmed digestion mix, and digested for 15 min at 37 °C. The microvessel pellet was again centrifuged at 800× *g*/10 min at 4 °C and washed with a culture medium containing serum. The microvessels were cultured in DMEM-F12 medium (PAA Laboratoris, Germany) containing 15% plasma-derived serum (PDS) (First Link, Birmingham, UK), 2 mM L-glutamine (GE Healthcare, Buckinghamshire, UK), BME vitamins (Sigma–Aldrich, USA), heparin (Sigma-Aldrich, USA), and 3 µM puromycine (Sigma-Aldrich, USA). After 7 days, cells were seeded on the upper chamber (apical compartment) of Transwell insert with a 0.4 µm pore size (Becton Dickinson, Franklin Lakes, NJ, USA) pre-coated with 10 µg/cm^2^ collagen type IV (Sigma-Aldrich, USA) and 5 µg/cm^2^ fibronectin (Sigma-Aldrich, USA). Endothelial cells were cultivated together with mixed glial cells cultivated as an adherent monolayer in the basolateral compartment of 12-well Cortar plates for further seven days in EBM-2 medium containing 15% PDS, 2 mM L-glutamine, BME vitamins, and BulletKit SingleQuots (Lonza, Slough, UK). After seven days, the inserts were ready for the experiments.

### 4.9. Permeability Assay

For the permeability experiments, only inserts with TEER values higher than 300 Ω·cm^2^ were used. Transwell inserts were washed with Ringer-HEPES buffer (150 mM NaCl, 5.2 mM KCl, 2.2 mM CaCl_2_, 0.2 mM MgCl_2_·6H_2_O, 6 mM NaHCO_3_, 5 mM HEPES, 2.8 mM glucose; pH 7.4). KYNA-1 was dissolved in Ringer-HEPES and applied to the upper (apical) compartment at a final concentration of 1 mg mL^−1^. All incubations were performed at 37 °C. Samples were collected from the basolateral side after 5, 15, 30, and 60 min. The concentrations of GA in the abluminal and luminal compartments were measured using a validated LC-MS/MS method. The permeability values of the inserts (PSf, for inserts with a coating only) and the insert plus endothelium (PSt, for inserts with a coating and cells) were considered by applying the following equation:1/PSe = 1/PSt − 1/PSf. To obtain the endothelial permeability coefficient (Pe, in cm·s^−1^), the permeability value (PSe) corresponding to the endothelium alone was divided by the porous membrane surface area of the insert.

### 4.10. Statistical Analysis

Statistical analyses were performed using GraphPad Prism 8.0.1 (GraphPad Software, Inc., San Diego, CA, USA). All data were subjected to statistical analysis based on their normal distribution, utilizing the appropriate statistical tests as chosen. Results are presented as mean values with accompanying standard deviations (SD) to indicate the variation.

For quantifying metabolite concentrations and conducting quality assessments, the MetIQ software (https://biocrates.com/webidq/ accessed on 16 June 2024) package was employed (BIOCRATES Life Sciences AG, Innsbruck, Austria). The internal standards served as a reference for metabolite concentration calculations. Univariate (*t*-tests and ANOVA) and multivariate statistics (partial least squares-discrimination analysis PLS-DA), as well as variable importance in projection (VIP) plots, were performed using MetaboAnalyst 5.0 (Xia Lab @ McGill, St. Anne de Bellevue, QC, Canada). Cross-validation of the PLS-DA classification applied five components for selection as the optimal number of components. The LOOCV cross-validation method was used. The performance of measurements, such as Accuracy, R2, and Q2, were considered. The VIP score was measured using the PLS-DA method. The VIP score is a measure of the importance of a feature in the PLS-DA model. This summarizes the contribution of a feature to the model. The VIP score of a feature was calculated as the weighted sum of the PLS weights. The PLS weight is the squared correlation between PLS-DA components and the original feature.

## 5. Conclusions

The stability of GA in various conditions and its demonstrated safety, with no observed toxicity in blood or liver, make it a promising compound for further research. It binds to HSA, potentially facilitating its transportation to target tissues. The lack of influence on body weight gain and water and fluid intake further supports its safety profile. The observed changes in behavior indicate the interesting potential of GA in neurological disorders, possibly influenced by its ability to permeate the blood–brain barrier and stimulation the proliferation of hippocampal progenitors in hilus and SGZ. These findings suggest that GA warrants further investigation for its therapeutic potential in neurological conditions.

## Figures and Tables

**Table 1:** Gyrophoric acid (GA) binding to human serum albumin (HSA).

	HSA		GA-HSA	
	1st Peak	2nd Peak	1st Peak	2nd Peak
peak position (λex/λem) (nm)	280/342	230/335	280/340	230/342
relative intensity (IF)	192	295	113	59
Δλ	62	105	60	112

**Table 2:** Calculated quenching and binding parameters of the HSA–GA system.

Ligand	K_SV_ × 10^5^ (M^−1^)	k_q_ × 10^13^ (M^−1^ s^−1^)	^a^ R^2^	K_B_ × 10^6^ (M^−1^)	*n*	^a^ R^2^
HSA	4.679	4.679	0.99	1.788	1.143	0.993

^a^ R^2^ = correlation coefficient.

**Table 3:** Food and fluid intake during the experiment.

	Exp. Week	INT (m)	GA (m)	INT (f)	GA (f)
food intake (g)	6th	26.58 ± 2.83	25.61 ± 2.20	17.00 ± 0.73	16.10 ± 1.11
	11th	23.00 ± 6.39	22. 38 ± 1.83	16.58 ± 3.01	15.72 ± 2.90
fluid intake (mL)	6th	46.58 ± 9.40	44.41 ± 4.03	24.83 ± 0.55	26.00 ± 0.42
	11th	49.17 ± 5.84	48.27 ± 5.62	37.83 ± 8.40	35.42 ± 7.13

Daily food intake was measured in grams (g) and fluid intake in milliliters (mL). Data are expressed as mean ± SD. INT—healthy intact, GA—gyrophoric acid.

**Table 4:** Blood parameters of intact male and female Sprague-Dawley rats compared to male and female groups after gyrophoric acid administration.

		INT (m)	GA (m)	INT (f)	GA (f)
**WBC**	10^9^/L	16.10 ± 1.96	17.30 ± 1.60	11.57 ± 5.38	10.26 ± 2.22
**LYM**	10^9^/L	12.30 ± 1.36	12.93 ± 0.84	8.86 ± 4.24	7.96 ± 2.01
**MON**	10^9^/L	0.20 ± 0.08	0.03 ± 0.20 **	0.16 ± 0.13	0.06 ± 0.05 *
**GRA**	10^9^/L	2.60 ± 0.51	1.16 ± 0.79 **	2.53 ± 1.20	1.23 ± 0.25 **
**HCT**	%	43.63 ± 0.95	45.93 ± 0.87	32.90 ± 9.47	30.17 ± 5.42
**HGB**	g/L	161.70 ± 4.77	165.70 ± 4.35	118.70 ± 34.39	109.00 ± 22.27
**RBC**	10^12^/L	8.40 ± 0.27	8.31 ± 0.07	5.66 ± 1.77	5.01 ± 0.98
**PLT**	10^9^/L	496.00 ± 62.83	455.00 ± 65.53	112.70 ± 61.90	117.70 ± 72.41
**MPV**	fl	4.90 ± 0.15	4.93 ± 0.18	4.80 ± 0.31	5.00 ± 0.45

Data are expressed as mean ± SD. Signifiance vs. INT: * *p* < 0.05; ** *p* < 0.01. WBC, white blood cells; LYM, lymphocytes; MON, monocytes; GRA, granulocytes; HCT, hematocrit; HGB, hemoglobin; RBC, red blood cells; PLT, platelets; MPV, mean platelet volume; GA, gyrophoric acid.

**Table 5:** Selected biochemical parameters in healthy male and female Sprague-Dawley rats.

		INT (m)	GA (m)	INT (f)	GA (f)
**LDH**	µkat/L	23.83 ± 6.86	26.88 ± 5.26	17.28 ± 4.46	17.83 ± 3.12
**CK**	µkat/L	58.48 ± 32.94	57.20 ± 16.30	25.48 ± 8.30	33.70 ± 21.24
**ALT**	µkat/L	0.91 ± 0.19	0.75 ± 0.19 *	0.57 ± 0.90	0.35 ± 0.10 **
**ALP**	µkat/L	2.27 ± 0.23	2.18 ± 0.37	1.42 ± 0.54	1.28 ± 0.30
**T Bil**	µmol/L	0.58 ± 0.50	0.40 ± 0.58	0.52 ± 0.73	1.80 ± 1.90
**CA**	mmol/L	2.25 ± 0.06	2.25 ± 0.12	2.44 ± 0.06	2.43 ± 0.06
**Crea**	µmol/L	29.53 ± 6.30	22.27 ± 4.83 **	24.30 ± 2.40	18.58 ± 3.77 **
**Urea**	mmol/L	5.78 ± 0.81	4.74 ± 0.55 **	5.17 ± 0.72	4.10 ± 0.87 *

Data are expressed as mean ± SD. Signifikance vs. INT: * *p* < 0.05; ** *p* < 0.01. LDH, lactate dehydrogenase; CK, creatine kinase; ALT, alanine amino transferase; ALP, alkaline phosphatase; T Bil, total bilirubin; CA, calcium; Crea, creatinine, Urea, blood urea; GA, gyrophoric acid.

## Data Availability

The data are available after a request.

## Supplementary Materials

The following supporting information can be downloaded at: https://www.mdpi.com/article/10.3390/ijms25126782/s1.

## List of Abbreviations

ALP	alkaline phosphatase
ALT	alanine amino transferase
Arg	arginine
ARRIVE	checklist of recommendations to improve the reporting of research involving animals
Asp	aspartate
BME	basal medium eagle
BSA	bovine serum albumin
C	concentration
Ca	calcium
CA1	cornu Ammonis 1 zone
CaCl_2_	calcium chloride
CK	creatine kinase
CO_2_	carbon dioxide
Crea	creatinine
DMEM	Dulbecco’s modified eagle medium
DMSO	dimethylsulfoxide
DNA	deoxyribonucleic acid
DNase I	deoxyribonuclease I
EBM-2	endothelial basal medium
EPM	elevated plus-maze
ESI	electrospray ionization
FCS	fetal calf serum
GA	gyrophoric acid
GCL	granular cell layer
GRA	granulocytes
HCl	hydrochloric acid
HCT	hematocrit
HEPES	4-(2-hydroxyethyl)-1-piperazineethanesulfonic acid
HGB	hemoglobin
HSA	human serum albumin
INT	healthy intact control rats
K_3_EDTA	tripotassium ethylenediaminetetraacetic acid
KCl	potassium chloride
KYNA-1	kynurenic acid
LC-MS/MS	liquid chromatography-mass spectrometry
LDH	lactate dehydrogenase
LYM	lymphocytes
Met-SO	methionine-sulfoxide
MgCl_2_·6H_2_O	magnesium chloride hexahydrate
MME	zinc-dependent metalloprotease neprilysin
MON	monocytes
MPV	mean platelet volume
MS	mass spectrometry
NADPH	nicotinamide adenine dinucleotide phosphate
NaHCO_3_	sodium bicarbonate
NaOH	sodium hydroxide
NeuN	neuronal nuclei
OFT	open field test
PDB	protein data bank
PDS	plasma-derived serum
Pe	permeability coefficient
Phe	phenylalanine
pKa	acid dissociation constant
PLS-DA	partial least squares-discrimination analysis
PLT	platelets
PSe	permeability value
PTP1B	eukaryotic protein tyrosine phosphatase
RBC	red blood cells
SD	standard deviation
SGZ	subgranular zone
SRM	standard reference material
SVZ	subventricular zone
T Bil	total bilirubin
TEER	trans-epithelial electrical resistance
Trp	tryptophan
Tyr	tyrosine
UV-VIS	ultraviolet and visible spectroscopy
WBC	white blood cells

## References

[1] Crawford S., Ranković B. (2015). Lichens Used in Traditional Medicine. Lichen Secondary Metabolites.

[2] Simko P., Kisková T. (2022). Uncovering the anticancer potential of lichen secondary metabolites. J. Anal. Oncol..

[3] Paukov A., Teptina A., Ermoshin A., Kruglova E., Shabardina L. (2022). The Role of Secondary Metabolites and Bark Chemistry in Shaping Diversity and Abundance of Epiphytic Lichens. Front. For. Glob. Change.

[4] Barbero M., Artuso E., Prandi C. (2018). Fungal anticancer metabolites: Synthesis towards drug discovery. Curr. Med. Chem..

[5] Goga M., Elečko J., Marcinčinová M., Ručová D., Bačkorová M., Bačkor M., Mérillon J.M., Ramawat K. (2020). Lichen metabolites: An overview of some secondary metabolites and their biological potential. Co-Evolution of Secondary Metabolites.

[6] Ingelfinger R., Henke M., Roser L., Ulshöfer T., Calchera A., Singh G., Parnham M.J., Geisslinger G., Fürst R., Schmitt I. (2020). Unraveling the pharmacological potential of lichen extracts in the context of cancer and inflammation with a broad screening approach. Front. Pharmacol..

[7] Molnár K., Farkas E. (2010). Current results on biological activities of lichen secondary metabolites: A review. Z. Für Naturforschung C.

[8] Stanojković T., Ranković B. (2019). Investigations of lichen secondary metabolites with potential anticancer activity. Lichen Secondary Metabolites: Bioactive Properties and Pharmaceutical Potential.

[9] Mohammadi M., Bagheri L., Badreldin A., Fatehi P., Pakzad L., Suntres Z., van Wijnen A.J. (2022). Biological effects of gyrophoric acid and other lichen derived metabolites, on cell proliferation, apoptosis and cell signaling pathways. Chem.-Biol. Interact..

[10] Buçukoglu T.Z., Albayrak S., Halici M.G., Tay T. (2013). Antimicrobial and Antioxidant Activities of Extracts and Lichen Acids Obtained from Some *Umbilicaria* Species from Central Anatolia, Turkey. J. Food Process. Preserv..

[11] Lohézic-Le Dévéhat F., Legouin B., Couteau C., Boustie J., Coiffard L. (2013). Lichenic extracts and metabolites as UV filters. J. Photochem. Photobiol. B Biol..

[12] Nguyen K.-H., Chollet-Krugler M., Gouault N., Tomasi S. (2013). UV-protectant metabolites from lichens and their symbiotic partners. Nat. Prod. Rep..

[13] Kumar KC S., Müller K. (1999). Lichen metabolites. 2. Antiproliferative and cytotoxic activity of gyrophoric, usnic, and diffractaic acid on human keratinocyte growth. J. Nat. Prod..

[14] Correché E.R., Enriz R.D., Piovano M., Garbarino J., Gómez-Lechón M.J. (2004). Cytotoxic and apoptotic effects on hepatocytes of secondary metabolites obtained from lichens. Altern. Lab. Anim. ATLA.

[15] Burlando B., Ranzato E., Volante A., Appendino G., Pollastro F., Verotta L. (2009). Antiproliferative effects on tumour cells and promotion of keratinocyte wound healing by different lichen compounds. Planta Medica.

[16] Bačkorová M., Bačkor M., Mikeš J., Jendželovský R., Fedoročko P. (2011). Variable responses of different human cancer cells to the lichen compounds parietin, atranorin, usnic acid and gyrophoric acid. Toxicol. Vitr. Int. J. Publ. Assoc. BIBRA.

[17] Kello M., Goga M., Kotorova K., Sebova D., Frenak R., Tkacikova L., Mojzis J. (2023). Screening Evaluation of Antiproliferative, Antimicrobial and Antioxidant Activity of Lichen Extracts and Secondary Metabolites In Vitro. Plants.

[18] Kosanić M., Ranković B. (2011). Antioxidant and antimicrobial properties of some lichens and their constituents. J. Med. Food.

[19] Bačkorová M., Jendželovský R., Kello M., Bačkor M., Mikeš J., Fedoročko P. (2012). Lichen secondary metabolites are responsible for induction of apoptosis in HT-29 and A2780 human cancer cell lines. Toxicol. Vitr..

[20] Elečko J., Vilková M., Frenák R., Routray D., Ručová D., Bačkor M., Goga M. (2022). A Comparative Study of Isolated Secondary Metabolites from Lichens and Their Antioxidative Properties. Plants.

[21] Norouzi H., Azizi A., Gholami M., Sohrabi M., Boustie J. (2020). Chemotype variations among lichen ecotypes of *Umbilicaria aprina* as revealed by LC-ESI-MS/MS: A survey of antioxidant phenolics. Environ. Sci. Pollut. Res. Int..

[22] Ristić S., Ranković B., Kosanić M., Stanojković T., Stamenković S., Vasiljević P., Manojlović I., Manojlović N. (2016). Phytochemical study and antioxidant, antimicrobial and anticancer activities of *Melanelia subaurifera* and *Melanelia fuliginosa* lichens. J. Food Sci. Technol..

[23] Ureña-Vacas I., González-Burgos E., Divakar P.K., Gómez-Serranillos M.P. (2022). Lichen Extracts from Cetrarioid Clade Provide Neuroprotection against Hydrogen Peroxide-Induced Oxidative Stress. Molecules.

[24] Ibrahim N., Ibrahim H., Kim S., Nallet J.-P., Nepveu F. (2010). Interactions between antimalarial indolone-N-oxide derivatives and human serum albumin. Biomacromolecules.

[25] Zhang Y., Shi S., Chen X., Zhang W., Huang K., Peng M. (2011). Investigation on the interaction between ilaprazole and bovine serum albumin without or with different C-ring flavonoids from the viewpoint of food–drug interference. J. Agric. Food Chem..

[26] Tabassum S., Ahmad M., Afzal M., Zaki M., Bharadwaj P.K. (2014). Synthesis and structure elucidation of a copper (II) Schiff-base complex: In vitro DNA binding, pBR322 plasmid cleavage and HSA binding studies. J. Photochem. Photobiol. B Biol..

[27] Zhang G., Wang L., Pan J. (2012). Probing the binding of the flavonoid diosmetin to human serum albumin by multispectroscopic techniques. J. Agric. Food Chem..

[28] Lakowicz J.R. (2006). Principles of Fluorescence Spectroscopy.

[29] Topală T., Bodoki A., Oprean L., Oprean R. (2014). Bovine serum albumin interactions with metal complexes. Clujul Med..

[30] Feroz S.R., Mohamad S.B., Bujang N., Malek S.N., Tayyab S. (2012). Multispectroscopic and molecular modeling approach to investigate the interaction of flavokawain B with human serum albumin. J. Agric. Food Chem..

[31] Li X., Chen D., Wang G., Lu Y. (2013). Study of interaction between human serum albumin and three antioxidants: Ascorbic acid, α-tocopherol, and proanthocyanidins. Eur. J. Med. Chem..

[32] Schellman J.A. (1997). Temperature, stability, and the hydrophobic interaction. Biophys. J..

[33] Wani T.A., Bakheit A.H., Al-Majed A.-R.A., Bhat M.A., Zargar S. (2017). Study of the interactions of bovine serum albumin with the new anti-inflammatory agent 4-(1, 3-Dioxo-1, 3-dihydro-2 H-isoindol-2-yl)-N′-[(4-ethoxy-phenyl) methylidene] benzohydrazide using a multi-spectroscopic approach and molecular docking. Molecules.

[34] Alsaif N.A., Wani T.A., Bakheit A.H., Zargar S. (2020). Multi-spectroscopic investigation, molecular docking and molecular dynamic simulation of competitive interactions between flavonoids (quercetin and rutin) and sorafenib for binding to human serum albumin. Int. J. Biol. Macromol..

[35] Huang S., Qiu H., Lu S., Zhu F., Xiao Q. (2015). Study on the molecular interaction of graphene quantum dots with human serum albumin: Combined spectroscopic and electrochemical approaches. J. Hazard. Mater..

[36] Hale M.E. (1956). 2,4-Dihydroxy depsides in North American lichens. Trans. Kans. Acad. Sci..

[37] Yang F., Zhang Y., Liang H. (2014). Interactive association of drugs binding to human serum albumin. Int. J. Mol. Sci..

[38] Simko P., Leskanicova A., Suvakova M., Blicharova A., Karasova M., Goga M., Kolesarova M., Bojkova B., Majerova P., Zidekova N. (2022). Biochemical Properties of Atranorin-Induced Behavioral and Systematic Changes of Laboratory Rats. Life.

[39] Urbanska N., Simko P., Leskanicova A., Karasova M., Jendzelovska Z., Jendzelovsky R., Rucova D., Kolesarova M., Goga M., Backor M. (2022). Atranorin, a secondary metabolite of lichens, exhibited anxiolytic/antidepressant activity in Wistar rats. Life.

[40] Araújo H.D.A.d., Silva H.A.M.F., Silva Júnior J.G.d., Albuquerque M.C.P.d.A., Coelho L.C.B.B., Aires A.d.L. (2021). The Natural Compound Hydrophobic Usnic Acid and Hydrophilic Potassium Usnate Derivative: Applications and Comparisons. Molecules.

[41] Yin L.K., Tong K.S. (2009). Elevated Alt and Ast in an Asymptomatic Person: What the primary care doctor should do?. Malays. Fam. Physician.

[42] Park J.H., Choi J., Jun D.W., Han S.W., Yeo Y.H., Nguyen M.H. (2019). Low alanine aminotransferase cut-off for predicting liver outcomes; a nationwide population-based longitudinal cohort study. J. Clin. Med..

[43] Carrillo J., Howard E.C., Moten M., Houck B.D., Czachowski C.L., Gonzales R.A. (2008). A 3-day exposure to 10% ethanol with 10% sucrose successfully initiates ethanol self-administration. Alcohol.

[44] Kisková T., Ekmekcioglu C., Garajová M., Orendáš P., Bojková B., Bobrov N., Jäger W., Kassayová M., Thalhammer T. (2012). A combination of resveratrol and melatonin exerts chemopreventive effects in N-methyl-N-nitrosourea-induced rat mammary carcinogenesis. Eur. J. Cancer Prev..

[45] Chen W., Wang Z., Ma C., Ma X., Meng W., Yin F., Yang Y. (2023). Tactile cues are important to environmental novelty during repeated open field tests. Behav. Process..

[46] Sturman O., Germain P.-L., Bohacek J. (2018). Exploratory rearing: A context-and stress-sensitive behavior recorded in the open-field test. Stress.

[47] Ortman H.A., Newby M.L., Acevedo J., Siegel J.A. (2021). The acute effects of multiple doses of methamphetamine on locomotor activity and anxiety-like behavior in adolescent and adult mice. Behav. Brain Res..

[48] Rojas-Carvajal M., Fornaguera J., Mora-Gallegos A., Brenes J.C. (2018). Testing experience and environmental enrichment potentiated open-field habituation and grooming behaviour in rats. Anim. Behav..

[49] Campos-Cardoso R., Novaes L.S., Godoy L.D., Dos Santos N.B., Perfetto J.G., Lazarini-Lopes W., Garcia-Cairasco N., Padovan C.M., Munhoz C.D. (2023). The resilience of adolescent male rats to acute stress-induced delayed anxiety is age-related and glucocorticoid release-dependent. Neuropharmacology.

[50] Beaman E.E., Bonde A.N., Larsen S.M.U., Ozenne B., Lohela T.J., Nedergaard M., Gíslason G.H., Knudsen G.M., Holst S.C. (2023). Blood–brain barrier permeable β-blockers linked to lower risk of Alzheimer’s disease in hypertension. Brain.

[51] Bickel U. (2022). Modeling blood–brain barrier permeability to solutes and drugs in vivo. Pharmaceutics.

[52] Latacz G., Lubelska A., Jastrzębska-Więsek M., Partyka A., Marć M.A., Satała G., Wilczyńska D., Kotańska M., Więcek M., Kamińska K. (2019). The 1,3,5-triazine derivatives as innovative chemical family of 5-HT6 serotonin receptor agents with therapeutic perspectives for cognitive impairment. Int. J. Mol. Sci..

[53] Studzińska-Sroka E., Majchrzak-Celińska A., Zalewski P., Szwajgier D., Baranowska-Wójcik E., Żarowski M., Plech T., Cielecka-Piontek J. (2021). Permeability of *Hypogymnia physodes* extract component—Physodic acid through the blood–brain barrier as an important argument for its anticancer and neuroprotective activity within the central nervous system. Cancers.

[54] Di L., Kerns E.H., Li S.Q., Petusky S.L. (2006). High throughput microsomal stability assay for insoluble compounds. Int. J. Pharm..

[55] Chatterjee T., Pal A., Dey S., Chatterjee B.K., Chakrabarti P. (2012). Interaction of virstatin with human serum albumin: Spectroscopic analysis and molecular modeling. PLoS ONE.

[56] Petitpas I., Petersen C.E., Ha C.-E., Bhattacharya A.A., Zunszain P.A., Ghuman J., Bhagavan N.V., Curry S. (2003). Structural basis of albumin–thyroxine interactions and familial dysalbuminemic hyperthyroxinemia. Proc. Natl. Acad. Sci. USA.

[57] Kertys M., Grendar M., Horak V., Zidekova N., Skalnikova H.K., Mokry J., Halasova E., Strnadel J. (2021). Metabolomic characterisation of progression and spontaneous regression of melanoma in the melanoma-bearing Libechov minipig model. Melanoma Res..

[58] Kertys M., Grendar M., Kosutova P., Mokra D., Mokry J. (2020). Plasma based targeted metabolomic analysis reveals alterations of phosphatidylcholines and oxidative stress markers in guinea pig model of allergic asthma. Biochim. Biophys. Acta (BBA)-Mol. Basis Dis..

[59] Majerova P., Hanes J., Olesova D., Sinsky J., Pilipcinec E., Kovac A. (2020). Novel blood–brain barrier shuttle peptides discovered through the phage display method. Molecules.

[60] Pardridge W.M. (2005). The blood-brain barrier: Bottleneck in brain drug development. NeuroRx.

