# Supplementary material for: The First In Vivo Study Shows That Gyrophoric Acid Changes Behavior of Healthy Laboratory Rats
Source: Int J Mol Sci. 2024 Jun 20;25(12):6782. doi: 10.3390/ijms25126782 (PMC11203575; doi:10.3390/ijms25126782)
Supplement: Supplementary file 1 [file ijms-25-06782-s001.zip › ijms-2985578-supplementary.pdf]

The First In Vivo Study Shows That Gyrophoric Acid Changes Behavior of Healthy Laboratory Rats

Patrik Simko [1], Andrea Leskanicova [1], Maria Suvakova-Nunhart [2], Jan Koval [1], Nela Zidekova [3], Martina Karasova [4], Petra Majerova [5], Ludmila Verboova [6], Alzbeta Blicharova [6], Michal Goga [1], Martin Kertys [3], Ivan Barvik [7], Andrej Kovac [5] and Terezia Kiskova [1,*]

**OFT**	INT (m)	GA (m)	INT (f)	GA (f)
rearing	7.83 ± 8.61	19.17 ± 7.50 **	22.16 ± 9.60	35.83 ± 8.42 **
washing	5.16 ± 3.13	4.83 ± 2.48	2.53 ± 1.42	1.17 ± 0.10
defecation	0.00 ± 0.00	0.17 ± 0.41	0.53 ± 0.22	0.50 ± 0.84
time in center	12.10 ± 27.22	14.02 ± 20.05	13.83 ± 3.31	13.27 ± 1.97
time on periphery	359.88 ± 11.06	373.74 ± 25.33	362.22 ± 24.58	377.25 ± 28.01
traveled distance	333.25 ± 144.82	345.90 ± 98.20	366.55 ± 114.01	365.17 ± 125.24
average speed	0.81 ± 0.42	0.78 ± 0.11	0.99 ± 0.41	1.04 ± 0.51
**EPM**	INT (m)	GA (m)	INT (f)	GA (f)
rearing	19.50 ± 6.22	29 ± 4.10 **	20.5 ± 2.35	29.83 ± 1.47 **
washing	2.33 ± 1.51	2.66 ± 1.75	2.00 ± 1.41	1.67 ± 0.82
defecation	1.17 ± 0.75	1.00 ± 1.10	0.33 ± 0.52	0.50 ± 1.22
center crossings	6.17 ± 1.40	11.67 ± 4.16 ***	4.00 ± 2.61	11.83 ± 5.00 **
time in open arms	12.17 ± 5.98	31.83 ± 14.99 ***	17.17 ± 15.61	27.17 ± 7.44 **

Table S1. **Open field test.** Data are expressed as mean ± SD. Significance vs. INT is shown as ** p < 0.01. Rearing and washing activities as well as defecation boluses are expressed as counts, time in the center and time on the periphery are expressed in seconds, traveled distance is given in meters, and average speed in m.s-1. m – male, f – female

Elevated plus maze. Data are expressed as mean ± SD. Significance vs. INT is shown as ** p < 0.01 and *** p < 0.001. Rearing and washing activities, defecation boluses as well as center crossings are expressed as counts, and time spent in open arms is expressed in seconds. m – male, f - female
